# Robot-assisted thoracic surgery for benign tumors at the cervicothoracic junction: a propensity-matched study

**DOI:** 10.1038/s41598-024-54653-1

**Published:** 2024-02-21

**Authors:** Maierhaba Maitiyasen, Hao Peng, Yvxuan Liu, Jingfeng Li, Chuan Gao, Jing Chen, Jun Yi

**Affiliations:** 1 Department of Cardiothoracic Surgery, Jinling Hospital, Affiliated Hospital of Medical School, Nanjing University, 305 East Zhongshan Road, Nanjing, Jiangsu People’s Republic of China; 2 Department of Cardiothoracic Surgery, Jinling Hospital, Nanjing University of Chinese Medicine, Nanjing, Jiangsu People’s Republic of China

**Keywords:** Surgical oncology, Quality of life

## Abstract

This study aimed to assess the feasibility and safety of robot-assisted thoracic surgery (RATS) for resecting benign tumors of the cervicothoracic junction. Between 2017 and 2021, a total of 54 patients with benign cervicothoracic junction tumors were included. Among them, 46 underwent RATS while 8 underwent open surgery. Using a propensity score based on four variables (age, sex, comorbidity, and tumor size). The outcomes compared included short-term outcomes such as blood loss, as well as long-term outcomes including respiratory function and patients' postoperative health-related quality of life. No operative deaths occurred in this study. RATS was associated with less intraoperative blood loss (102 < 380 ml, P = 0.001) and a shorter length of hospital stay (1.8 < 4.8, P < 0.001). After a median follow-up of 37 months, no recurrences were reported, and no statistically significant differences were found in the 3-year survival between the two groups. The postoperative respiratory function of patients with open surgery showed a significant decrease compared to preoperative levels and were lower than those of RATS patients. In terms of health-related quality of life, RATS was associated with a better mean EQ-5D-5L index than open surgery (0.808 > 0.650, P < 0.05). In RATS, tumor sizes > 5 cm (mean ± SD = 0.768 ± 0.111, P = 0.028) and neurogenic tumors (mean ± SD = 0.702 ± 0.082, P < 0.001) remained significantly and independently associated with a lower EQ-5D-5L index. This study demonstrated that robot-assisted thoracic surgery for benign tumors of the cervicothoracic junction is a safe and technically feasible procedure, particularly for tumors < 5 cm and non-neurogenic tumors.

## Introduction

The cervicothoracic junction (CTJ) is a complex region connecting the neck and thoracic cavity, spanning from the seventh cervical vertebra to the fourth thoracic vertebra^1^. Its intricate anatomy and location require specialized expertise from spinal, thoracic, and vascular surgeons, particularly cardiothoracic surgeons, who face significant challenges in the thoracic inlet, superior mediastinum, and upper thoracic cavity. Although rare, benign tumors originating from the CTJ can cause significant pain, discomfort, and mobility issues, thereby affecting a patient’s quality of life. Surgical resection is generally the most effective treatment option, with the main objective being complete tumor removal (R0 resection) while preserving the function and structure of the surrounding nerves and blood vessels^2^.

Various surgical approaches have been developed to ensure the complete removal of CTJ tumors. The posterior Shaw-Paulson approach^3^ was first used to remove superior sulcus tumors and has since been applied to other diseases with less invasive techniques. Including the trans-sternal approach, “trap-door” thoracotomy, hemi-clamshell approach, and transmanubrial osteomuscular sparing approach (TMA), these techniques are still highly invasive and have significant postoperative recovery times^4,5^. Minimally invasive techniques such as video-assisted thoracic surgery (VATS) and robot-assisted thoracic surgery (RATS) have shown comparable effectiveness to open thymectomy with fewer complications, shorter hospital stays, and shorter operative times^6^. However, VATS has limitations in terms of visualization and instrument flexibility. RATS has gained popularity over the past decade for the removal of various mediastinal masses, and studies have shown its safety and effectiveness^7^. The robotic system provides high-definition three-dimensional views, up to 10 times image magnification, seven degrees of freedom of surgical instruments, and filtration of physiologic hand tremors, making it ideal for removing tumors in a small space^8^. Compared with traditional open surgical procedures, RATS offers increased precision, improved visualization, reduced blood loss, and faster recovery times^9^.

Few studies have examined the benefits of robotic technology for the treatment of CTJ tumors. While some studies have examined the advantages of RATS for mediastinal tumors^10^, there are limited studies on its application in CTJ tumors. In this study, we evaluated the short-term effectiveness and long-term impact on quality of life of RATS for the treatment of benign CTJ tumors, assessing its safety and effectiveness.

## Methods

Between April 2017 and December 2021, a total of 54 patients with benign cervicothoracic junction tumors were included. Among them, 46 patients with benign tumors at the CTJ underwent robot-assisted thoracic surgery using the da Vinci S HD system (Intuitive Surgical Inc., Sunnyvale, CA, USA). 8 patients underwent open surgery include trans-sternal approach, hemi-clamshell approach and supraclavicular approach. Preoperative diagnosis of a benign tumor at the CTJ was determined based on clinical symptoms and thoracic computed tomography. Informed consent to undergo lesion excision with the da Vinci robot-assisted procedure was obtained from all patients.

**Figure 1:**
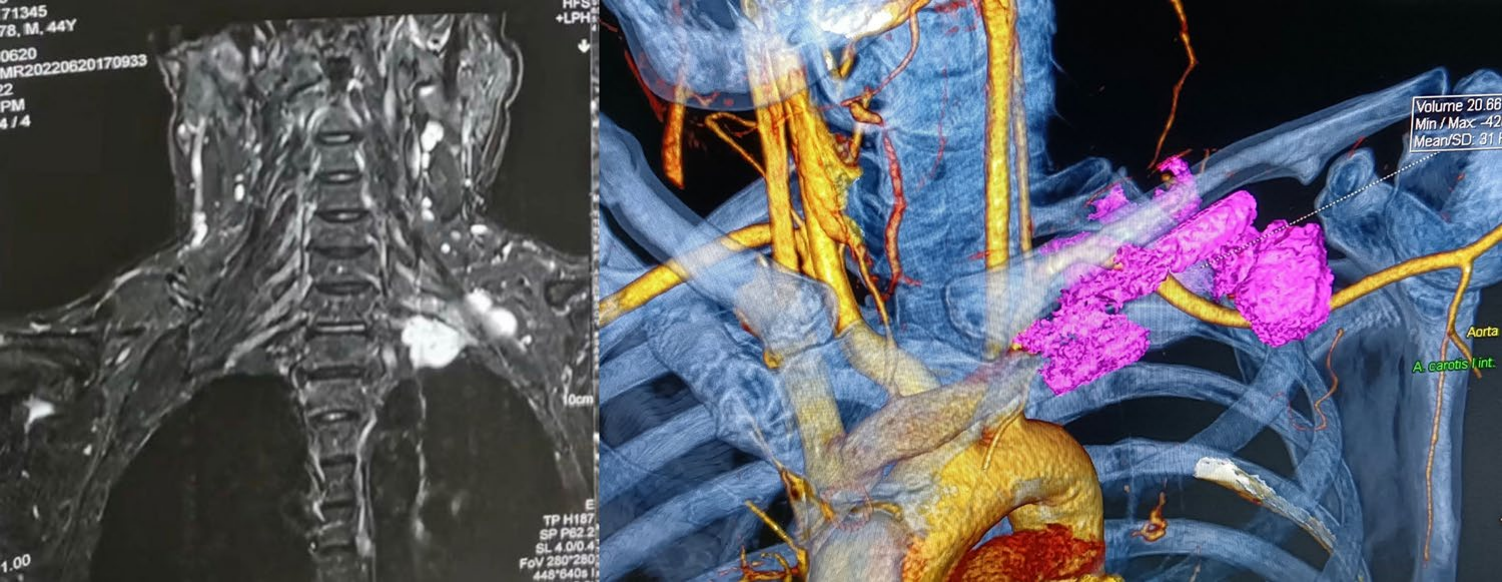
Three-dimensional reconstruction of cervicothoracic junction tumor.

Cardiopulmonary function and tumor resectability were evaluated before surgery. Chest-enhanced CT was performed to assess the tumor location and capsule integrity. For large tumors or those with significant external invasion, 3D tumor reconstruction may be performed to assess the possibility of complete resection (Fig. 1).

### Ethical approval

Our study was approved by the Institutional Review Board of the Jinling Hospital Ethics Committee, in compliance with the Declaration of Helsinki.

### Consent to participate

This study was retrospective and approved by the Ethics Committee of Jinling Hospital. The exemption from the Informed Consent Form was approved by the Ethics Committee of Jinling Hospital.

### Surgical techniques

The CTJ has three parts: the front, middle, and back, separated by the attachment point of the scalene muscles to the first rib^11^. The anterior compartment is bordered by the sternum and is posterior to the middle scalene muscle. The middle compartment lies between the anterior and the middle scalene muscles. The surgical approach and patient position depend on the location of the tumor. For anterior CTJ tumors, the patient was placed in a 30-degree semi-supine position, like traditional thymectomy.

**Figure 2:**
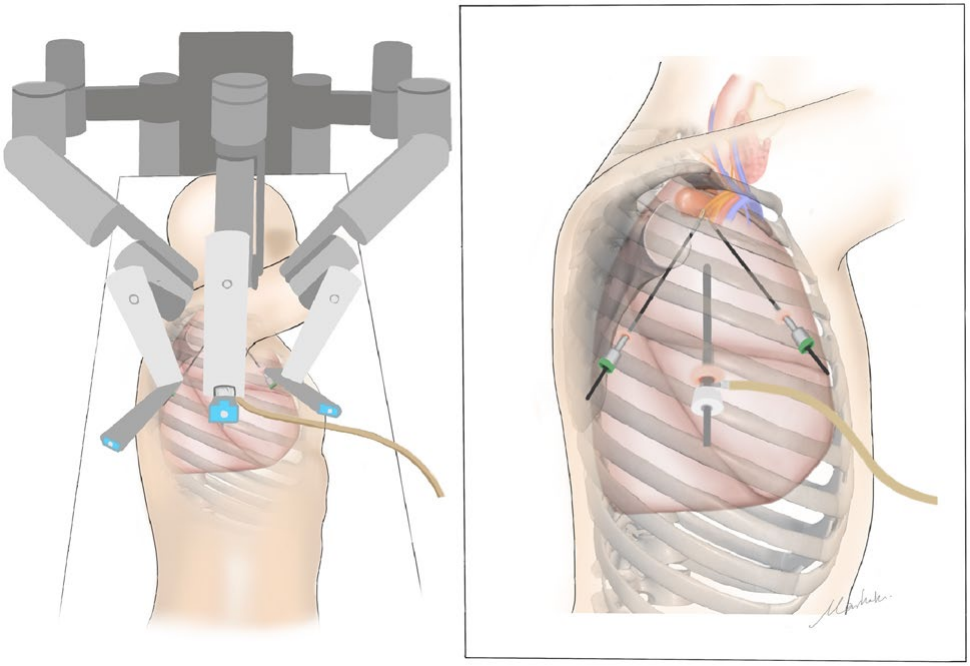
Schematic illustration of robot-assisted thoracic surgery for cervicothoracic junction tumor.

Surgical techniques for tumors located at the middle and posterior cervicothoracic junction via RATS involve placing patients in a lateral position under general anesthesia with double-lumen endotracheal intubation for single-lung ventilation. The procedure uses three ports without the need for an assistant port. A 12 mm trocar was placed for the camera port in the 6th intercostal space at the lower border of the latissimus dorsi muscle, while an 8 mm trocar for robotic arm 1 was placed in the 4th intercostal space at the anterior axillary line, and another 8 mm trocar for robotic arm 2 was placed in the 6th intercostal space at the scapular lines. The use of fenestrated forceps for the left arm and a permanent cautery hook for the right arm was preferred with the robotic endoscope positioned between the robotic arms. During operation, the 30° camera can be adjusted either up or down (Fig. 2) (Video 1).

Subsequently, the da Vinci robot system was connected, and carbon dioxide (8–10 mmHg) was insufflated into the thoracic cavity. The procedure starts with a comprehensive exploration of the cavity to identify the tumor’s borders, fatty tissue, and its relationship with the nerves and blood vessels. Dissection begins at the outer edge of the tumor, and blunt dissection along the surface close to the mass is recommended to free the capsule and ensure complete tumor removal while avoiding damage to the nerves and thyroid. Harmonic curves are preferred over electric scalpels to prevent heat injury to the nerves. For the subclavian artery or vein branches, conventional stapler devices, such as hem-lock clips, may be used, or cautery hooks can electrocauterize small branches. The left arm protects the nerves and blood vessels during dissection, while the right arm separates the deep part of the tumor by gently lifting with grasping forceps.

The specimen was removed from the port of the right arm using an Endo bag (Kang Ji, China). After the mediastinum was carefully inspected for any remaining tumor tissue and hemostasis was achieved, we placed a 20 Fr draining tube into the thoracic cavity through the endoscopic incision.

### Follow up

Safety assessments included identification of treatment-related complications within 30 days of surgery. Complications were categorized according to the Clavien–Dindo classification system^12^.

The patients were closely monitored after treatment. Follow-up appointments were scheduled every 3 months during the first year and every 6 months thereafter. The patients underwent physical examination, chest radiography, and blood tests at each visit, and chest scans were required annually for the first 3 years postoperatively, unless otherwise indicated. Survival was measured from the first day of surgery until death or loss to follow-up, and event-free survival was measured from the first day of surgery until any adverse events occurred, such as tumor progression or recurrence.

To address the limitation of being unable to collect preoperative quality of life questionnaire data in the retrospective study, we introduced pre- and postoperative pulmonary function data. The postoperative pulmonary function data was the most recent examination results for the patients, which may provide insight into long-term prognosis.

### EQ-5D-5L questionnaire

The EQ-5D-5L questionnaire, consisting of a descriptive system and a visual analog scale (EQ VAS), was used to assess patients’ long-term postoperative quality of life. The descriptive system included five dimensions [morbility (MO), self-care (SC), usual activity (UA), pain/discomfort (PD), anxiety/depression (AD)]. Each dimension has five potential levels: from ‘1’ (no limitations) to ‘5’ (extreme limitations). The EQ-5D-5L descriptive system defines 3125 potential different health states. Each health state can be assigned a single number representing the overall health score—the EQ-5D-5L Index. In the present study, to estimate EQ-5D-5L Index values, an EQ-5D-5L value set for China obtained using direct measurement methods (time trade-off, discrete choice experiment) in the Chinese population was used^13^. The EQ-5D-5L value set for China range from − 0.391 to 1.0 (full health). No disease-specific instruments were used in this study.

The interviewers collected data during face-to-face interviews (August to December 2022). The EQ-5D-5L questionnaire was distributed as a paper-and-pencil version and official Chinese translations were used to reduce errors.

### Statistical analysis

Baseline demographics, clinical characteristics, forced vital capacity (FVC), forced expiratory volume per second (FEV1) and health-related quality of life (HRQoL) scores are presented as the mean ± SD for continuous outcomes and frequencies (percentage) for dichotomous outcomes. The Shapiro–Wilk test was used to examine the parametricity of the distribution. Differences in outcomes between the 2 surgical approaches were assessed using a propensity score (PS)-matched analysis: Based on the PS, patients undergoing RATS versus Open surgery were matched with the nearest neighbour method without replacement. PS was estimated using a priori selected variables that have been associated with the likelihood of RATS: age, gender, comorbidity, and tumor size. After matching, outcomes were evaluated with the same methods described previously. Linear regression models were used to assess the associations between participant characteristics and the EQ-5D-5L Index. Mann–Whitney and ANOVA tests were used to evaluate statistical significance (P < 0.05). Univariable models were used to evaluate associations, and Chi-squared tests were used to assess associations between predictor variables. A final multivariable model was developed based on significant associations (P < 0.05, 95% CI that did not cross zero) in univariate models, excluding collinear variables. Survival analysis was performed using the Kaplan–Meier method, and the curves were compared using the log-rank test.

All analyses were performed using the IBM SPSS statistics software (version 22.0; IBM, Inc., Armonk, NY, USA).

### Ethics approval and consent to participate

Ethical approval was granted by the Ethics Committee of Jinling Hospital, Nanjing University (Jiangsu, China), and the requirement for patient consent was waived.

### Consent for publication

This article is a retrospective study, and all data were collected from our center's database with patients' consent.

## Results

### Baseline demographics and clinical characteristics

The demographic data of the two groups before and after case matching are presented in Table 1. In RATS group, all 46 patients (22 males and 24 females) with benign CTJ tumors underwent successful resection. RATS was performed in the lateral decubitus position in 29 (63.0%) patients with middle and posterior CTJ lesions and in the semi-lateral decubitus position in 17 patients who only required resection of anterior CTJ lesions. One case involved RATS exploration combined with a paravertebral incision, and the other involved RATS exploration combined with a transcervical incision. Most patients (35 cases, 76.1%) were diagnosed through asymptomatic examination, whereas the remaining patients (11 cases, 23.9%) exhibited various initial symptoms. Neck and shoulder pain (seven cases) was the most common presentation, followed by a palpable mass. The tumor sizes ranged between 1.5 × 2 cm and 12 × 11 cm, with a median of 5 cm used for binary classification.

**Table 1:** RATS versus open surgery: baseline characteristics and short-term outcome before and after propensity score matching.

	Before match (N = 54)			After match (N = 10)		
	RATS (N = 46)	Open (N = 8)	P-value	RATS (N = 5)	Open (N = 5)	P-value
Age (year), mean (SD)	44.6 (11.1)	51.0 (7.6)	0.122^a^	49 (9.67)	50 (9.19)	0.871^a^
Gender (male), n (%)	22 (47.8)	4 (50)	0.564^b^	3 (60)	3 (60)	1.000^b^
Comorbidity, n (%)	14 (30.4)	3 (37.5)	0.691^b^	1 (20)	1 (20)	1.000^b^
Symptoms (yes), n (%)	11 (23.9)	2 (25)	0.947^b^	1 (20)	1 (20)	1.000^b^
Tumor size, n (%)						
< 5 cm	25 (54.3)	1 (12.5)	0.029^b^	1 (20)	1 (20)	1.000^b^
≥ 5 cm	21 (45.7)	7 (87.5)		4 (80)	4 (40)	
Total operative time, mean [range] (min)	120.07 (55–200)	195.75 (115–325)	0.001^a^	138 (80–200)	224 (115–300)	0.045^a^
Total blood lost, mean [range] (ml)	125.98 (5–500)	562.5 (300–700)	0.000^a^	102 (20–200)	380 (200–500)	0.001^a^
Postoperative hospital stays, mean [range] (days)	2.5 (1–6)	6.13 (3–14)	0.000^a^	1.8 (1–2)	4.8 (4–6)	0.000^a^
Thoracic drainage tube removal, median [range] (days)	1.26 (1–5)	3.25 (3–4)	0.000^a^	1.0 (1)	3.2 (3–4)	0.000^a^
Postoperative complication, n (%)	4 (8.7)	3 (37.5)	0.025^b^	1 (20)	2 (40)	0.490^b^

^a^*t*-test.

^b^χ^2^ test or Fisher’s exact test.

In Open surgery group, 8 patients underwent open surgery, with procedures including the trans-sternal approach and hemi-clamshell approach. Among them, 2 patients presented with initial symptoms, including dyspnea and neck pain. Seven patients had tumors larger than 5 cm, with five undergoing a trans-sternal approach and two undergoing a hemi-clamshell approach. Only one patient with a tumor smaller than 5 cm underwent the supraclavicular incision.

PS matching was used to create a cohort of 10 patients (1:1 ratio), each of whom had a complete resection (R0) either through RATS or Open surgery. Before matching, compared to RATS group, the open surgery patients were more likely to present with larger tumors [> 5 cm, N = 7 (87.5%) vs N = 21 (45.7%); P = 0.029]. After matching, no significant differences were observed between the RATS and open surgery groups with regard to age, gender, comorbidity, tumor size (Table 1).

### Clinical outcome

In short-term outcomes, including operative time, intraoperative blood loss, length of hospital stays, and duration of chest tube removal, RATS demonstrated superior results compared to open surgery (Table 1). No deaths were reported within 30 days of surgery. There were no significant differences in postoperative complications between two groups. Among which the RATS group perioperative complication morbidity (grade I–II complications according to the Clavien–Dindo classification) occurred in four patients (8.7%) (Table 2). However, the postoperative complications following open surgery were more severe, including one patient with poor wound healing requiring secondary debridement and closure. Postoperative pathology and complications are detailed in Table 2.

**Table 2:** Postoperative pathology and complication of patients with cervicothoracic junction tumors.

Characteristics	RATS (N = 46)	Open (N = 8)
Histological identification n (%)		
Neurogenic	18 (39.1)	4 (50)
Schwannomas	10	1
Neurofibroma	5	1
Ganglioneuroma	3	2
Bronchogenic		
Bronchogenic cyst	14 (30.4)	
Thyroid		
Ectopic thyroid	3 (6.5)	2 (25)
Thymus		
Thymus cyst	6 (13.0)	
Other		
Vascular tumor	2 (4.5)	1 (12.5)
Mature cystic teratoma	3 (6.5)	1 (12.5)
Complications	RATS (N = 4)	Open (N = 3)
Horner syndrome	1	1
Hoarseness	1	0
Limb swelling	1	0
Limb numbness	1	1
Poor wound healing	0	1

In long-term outcomes, all 54 patients underwent complete resection (R0 resection) and were successfully followed up, with no recurrence reported during follow-up. The median follow-up time was 37 months, and 3-year survival rate of 100% and 5-year survival rate of 87.5%. Only one elderly patient died 56 months after surgery due to a heart attack. There was no significant difference in disease-free survival between the RATS and open surgery groups (P = 0.62).

**Figure 3:**
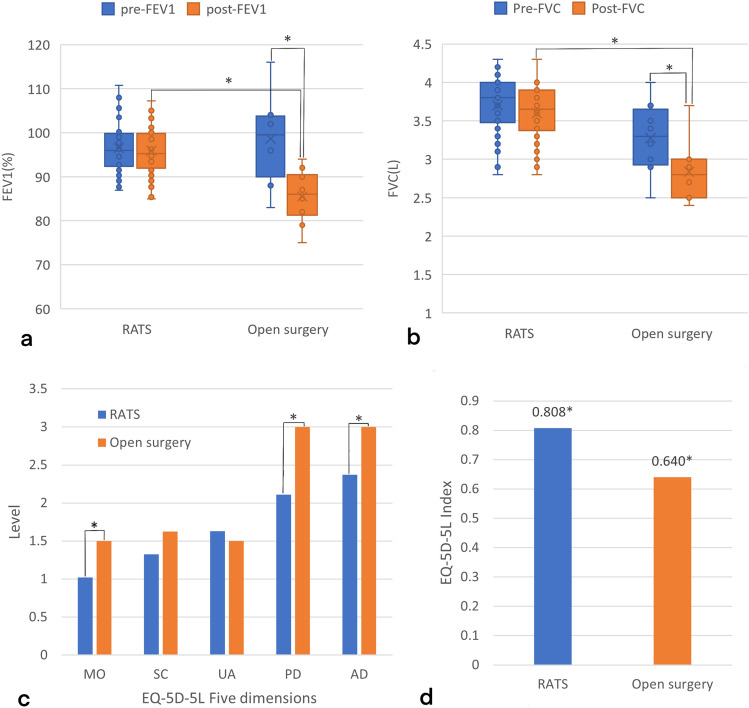
(**a**,**b**) Box-and-whisker plots of the median values and range of: (**a**) FEV1; (**b**) FVC before and after RATS and Open surgery over the study period. *P < 0.05. (**c**) The differences in the levels of the 5 dimensions of EQ-5D-5L between RATS and open surgery. (**d**) The differences in EQ-5D-5L index between RATS and open surgery.

The comparison of pre- and postoperative FEV1 and FVC between the two groups is shown in Fig. 3. The open surgery group demonstrated a significant decrease in postoperative respiratory function compared to the preoperative levels (FEV1: 98.6 > 85.5, P = 0.008; FVC: 3.27 > 2.65, P = 0.013), and also significantly lower than postoperative lung function of RATS group (FEV1: 85.5 < 95.9, P < 0.001; FVC: 3.59 > 2.81, P < 0.001). The comparison of postoperative quality of life between the two groups is shown in Fig. 3, with the EQ-5D-5L index for the open surgery group significantly lower than that of the RATS group (Index: 0.808 > 0.640, P = 0.002). As shown as Fig. 3, three dimensions of quality of life, including MO, self-care PD, and AD, RATS is significantly better than open surgery.

To explore the specific indications for RATS in benign CTJ tumors, we have presented the relationship between the EQ-5D-5L index and sociodemographic characteristics, as summarized in Table 3. In this group, the individual EQ-5D-5L index was normally distributed (W = 0.955, P = 0.073); nearly half of the participants had utility values of > 0.80.

**Table 3:** Results of linear regression models evaluating variables associated with EQ-5D-5L index of patients with cervicothoracic junction tumors in univariable models and a final multivariable model.

Characteristic	N (%)	EQ-5D-5L index				
		Mean (SD)	Univariable model		Multivariable model	
			β (95% CI)	P	β (95% CI)	P
Overall	46 (100)	0.808 (0.113)				
Gender						
Male	22 (47.8)	0.781 (0.118)	[Reference]			
Female	24 (52.2)	0.833 (0.105)	0.052 (− 0.015, 0.118)	0.124		
Age group						
< 60 years	32 (69.6)	0.812 (0.115)	[Reference]			
≥ 60 years	14 (30.4)	0.799 (0.113)	− 0.013 (− 0.087, 0.061)	0.724		
Tumor size						
< 5 cm	25 (54.3)	0.841 (0.106)	[Reference]		[Reference]	
≥ 5 cm	21 (45.7)	0.768 (0.111)	− 0.073* (− 0.138, − 0.008)	0.028	− 0.052* (− 0.990, − 0.040)	0.034
Tumor origin						
Neurogenic	18 (39.1)	0.702 (0.082)	− 0.197* (− 0.250, − 0.143)	< 0.001	− 0.182* (− 0.211, − 0.127)	< 0.001
Bronchogenic	14 (30.4)	0.899 (0.071)	[Reference]		[Reference]	
Thyroid	3 (6.5)	0.871 (0.062)	0.028 (− 0.123, 0.067)	0.561	− 0.021 (− 0.113, 0.070)	0.637
Thymus	6 (13.0)	0.820 (0.081)	0.028 (− 0.101, 0.045)	0.441	− 0.013 (− 0.084, 0.058)	0.707
Other	5 (11.0)	0.808 (0.025)	− 0.079* (− 0.157, − 0.001)	0.046	− 0.038 (− 0.122, 0.045)	0.356

*Coefficients with the statistical significance (P < 0.05).

Univariable models showed patients with tumors larger than 5 cm had a significantly lower mean index than those with tumors smaller than 5 cm (F = 5.173, P = 0.028). Similarly, the size of the tumor was negatively associated with utility value, meaning that patients with larger tumors had a lower health-related quality of life (HRQoL) on average. Patients with neurogenic tumors had a mean index that was significantly lower than that of patients with the other three types of tumors (F = − 16.143, P < 0.001). Our final multivariable model yielded results similar to those of univariate analyses. Tumor size and neurogenic tumors remained significantly independently associated, resulting in a decrease of 0.052 in the index for every one-unit increase in tumor size. The neurogenic nature of the cervicothoracic junction tumor reduced the outcome of the index by 0.182 (Table 3).

**Figure 4:**
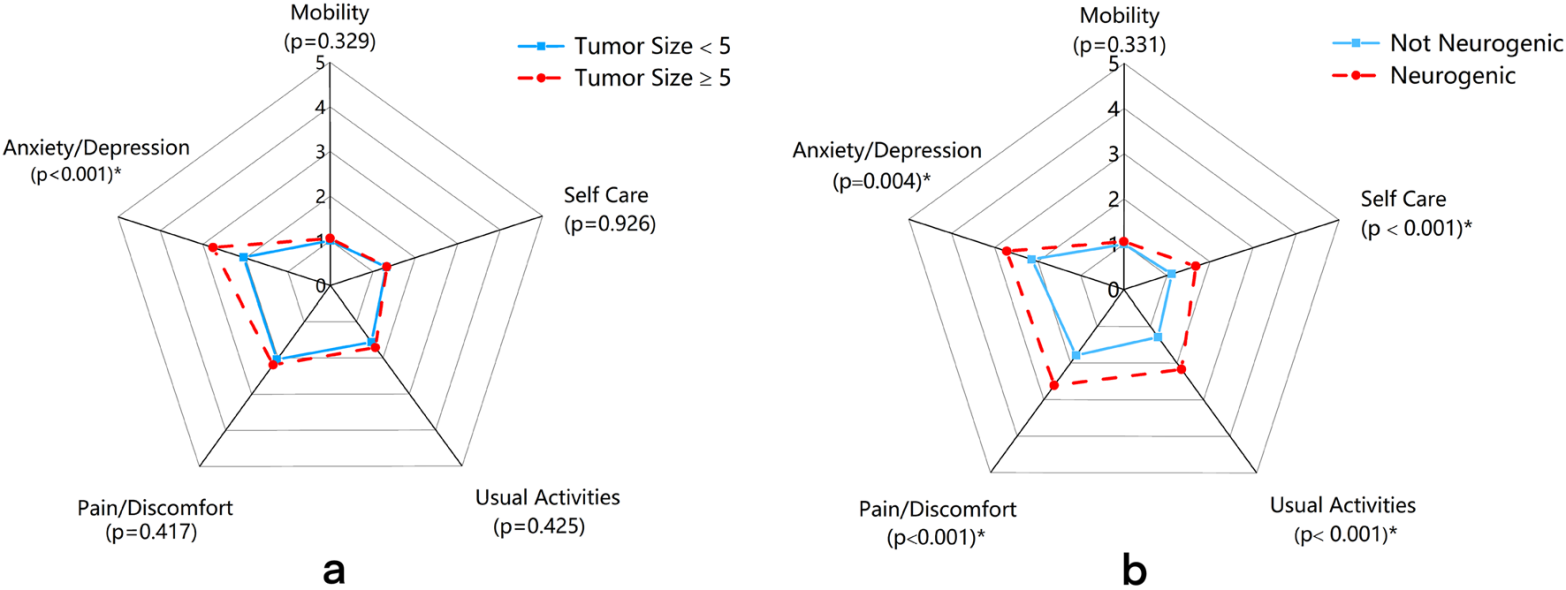
(**a**) Comparison between tumor size groups’ item scores for the five EQ-5D-5L items (*P < 0.05). (**b**) Comparison between Neurogenic groups’ item scores for the five EQ-5D-5L items (*P < 0.05). 1 is low symptom severity and 5 is high symptom severity.

A comparison of the levels of the five dimensions of the two subgroups is shown in Fig. 4. There were statistically significant differences between tumor size on AD [mean ± SD (tumor size ≥ 5 cm) = 2.76 ± 0.436 vs. (tumor size < 5 cm) = 2.04 ± 0.676; P < 0.001] (Fig. 3A). A comparison of the five dimensions’ levels between whether the tumor is neurogenic or not, there were statistically significant differences in SC [mean ± SD (neurogenic) = 1.67 ± 0.485 vs (not neurogenic) = 1.11 ± 0.315; P < 0.001], UA [mean ± SD (neurogenic) = 2.17 ± 0.514 vs (not neurogenic) = 1.29 ± 0.460; P < 0.001], PD [mean ± SD (neurogenic) = 2.61 ± 0.502 vs (not neurogenic) = 1.79 ± 0.418; P < 0.001], and AD [mean ± SD (neurogenic) = 2.72 ± 0.575 vs (not neurogenic) = 2.14 ± 0.651; P = 0.004] (Fig. 3B). Other domain-specific levels were similar.

## Discussion

Several approaches have been demonstrated for the management of the CTJ. With the development of minimally invasive techniques, the advantages of surgical machines in narrow areas such as the cervicothoracic junction have been self-evident; however, few studies have demonstrated and summarized the safety and efficacy of RATS management of tumors in this region. Based on our experience with 54 patients (RATS group = 46; open surgery group = 8), RATS is a safe and feasible approach. There were no conversions to open surgery because of complications during the procedure, and we obtained favorable results in terms of short-term outcomes.

Undoubtedly, different surgical approaches can provide different surgical perspectives for the resection of CTJ tumors originating from different sources (Supplementary Fig. 1). The selection of surgical approaches for CTJ tumors is complex, but recent advancements in RATS have streamlined this process. Both anterior and posterior CTJ tumors can be addressed using three intercostal ports, allowing surgeons to obtain an optimal surgical view by adjusting the robotic camera and arm angles during the procedure. For certain straightforward cases (The capsule is intact and does not involve vascular or nerves), as previously described in the surgical technique, it is sufficient to make only three small incisions without the requirement of an additional assistant port.

In certain complex cases where there is involvement of vessels or nerves (excluding the tumor involving large vessels that require vascular replacement) and the tumor is partially located close to the body surface, RATS can be enhanced by incorporating additional independent small incisions based on specific lesion locations. This approach enables minimally invasive surgery for complex CTJ tumors. We encountered a case of posterior dumbbell-shaped mediastinal schwannoma extending from the paravertebral to the thoracic cavity. In collaboration with spinal surgeons, and based on the patient's preoperative 3D reconstruction, we successfully excised the tumor using RATS exploration combined with a paravertebral incision. In another patient with a single neurofibroma measuring 6.5 × 4 cm, the lesion extended along the brachial plexus from the thoracic apex to the cervical root, surpassing the clavicle. We used RATS in conjunction with a transcervical incision to resect the tumor while preserving the nearby brachial plexus as much as possible. The lesion was successfully removed through R0 excision, leaving the patient with only three 1 cm wounds on the lateral chest wall and a 3 cm supraclavicular incision. Although the patient experienced numbness in the affected arm 30 days after surgery, it significantly improved after a year of treatment. No recurrence was observed during the 3-year follow-up period.

Tumors in the CTJ have diverse origins. Neurogenic tumors, including schwannomas, neurofibromas, and ganglioneuromas, were the most common tumor type in this study, accounting for 39.1% (18 cases). When managing benign neurogenic tumors at the CTJ, the primary goal is to achieve R0 resection, while maximizing neuroprotection. Several techniques, such as precise operative capabilities, energy-based instruments, and intraoperative nerve function monitoring, can help accomplish this goal. First, RATS is both safe and effective for well-exposed neurogenic tumors^14^, particularly in narrow areas such as the CTJ, where clear robotic visualization offers significant advantages^15^. The research findings regarding benign neurogenic tumors at the cervicothoracic junction also suggest that RATS significantly outperforms open surgery in terms of short-term outcomes and postoperative quality of life (Supplementary Table 1). We found that the lateral decubitus position resulted in the shortest mean operation time. Broussard et al. also demonstrated the efficacy of RATS for posterior mediastinal pathology when optimal trocar placement is employed^16^. Benign tumors with complete capsules can typically be entirely removed, with the main concern being to prevent thermal damage during resection. Ultrasonic devices are favored over electrosurgical energy instruments because of their reduced heat generation, which minimizes thermal damage to the surrounding tissues. Fascicular dissection can often be employed to remove neurofibromas, which involves extracting only the fascicle(s) entering and exiting the tumor mass, while preserving other peripheral fascicles. Nerve action potentials were monitored during the procedure to confirm the non-functional state of the affected fascicles, allowing for the removal of even large lesions such as neurofibromas as a single mass.

Additionally, the surgical learning curve also plays a critical role in surgical innovation and adoption, as it not only influences surgical outcomes but also affects patient safety and recovery times. The surgical technique for benign tumors at the CTJ by RATS is an extension of robot-assisted thymectomy. According to the experience of the lead surgeon in our center, the initial phase involves becoming familiar with robot-assisted thymectomy. As experience accumulates, the surgeon gradually advances to a higher stage, gaining further familiarity with the anatomy of the upper mediastinum. At this stage, the surgeon may become more proficient in performing the surgical procedure, leading to a gradual reduction in operation time and becoming acquainted with the surgical path selection for tumors at different sites at the CTJ. In the Plateau stage, the surgeon can skillfully determine the appropriate indications for RATS in the CTJ. The surgeons involved in this study believe that reaching the mastery stage involves the capacity to design personalized assisting incisions for complex cases, resulting in complete resection while reducing postoperative complication rates. Meanwhile, the study of the surgical learning curve is a specialized process, so further scientific data support is needed for the research on the learning curve of RATS for benign tumors at the CTJ.

Our study found no significant differences in mortality and recurrence rates between benign CTJ tumors. Consequently, we introduced the concept of health-related quality of life (HRQoL) to better evaluate long-term outcomes by using questionnaire surveys. Preference-based measures (PBM) are also employed to assess health status and quality of life^17^, and the EuroQol Five-Dimensional (EQ-5D) questionnaire is the most used generic PBM^18^. We assessed the patients' postoperative quality of life at least 1 year after surgery using the EuroQol Group's five-level EuroQol five-dimensional questionnaire (EQ-5D-5L). However, further responsiveness of EQ-5D-5L in RATS for CTJ tumors is required.

This study aimed to investigate the impact of sociodemographic characteristics on HRQoL in patients with benign CTJ tumors. We found that the mean EQ-5D-5L index for all patients was 0.808, with nearly half of the participants having utility values > 0.80, indicating generally good HRQoL. However, our study suggests that patients with larger tumors and neurogenic tumors were the main cause of lower postoperative HRQoL. Clinicians should be aware of the impact of tumor size and neurogenic origin on postoperative HRQoL and develop interventions to improve outcomes in high-risk patients.

After comparing subgroup levels across five dimensions, we found that chronic pain had the greatest impact on the index, primarily due to intercostal incision. Although pain intensity may not be severe, it can still reduce post-surgery quality of life, cause distress, and affect emotional well-being, potentially leading to anxiety and other symptoms. This may also account for the higher levels of anxiety/depression in larger tumors than in smaller tumors. Although most patients have the same surgical approach, we observed that for tumors larger than 5 cm, incisions are often extended to facilitate tumor removal. We deduced that this may be the reason why patients with tumors larger than 5 cm experience a higher proportion of chronic pain, consequently further affecting their Health-Related Quality of Life (HRQOL). Neurogenic tumors pose a higher risk of dimensions beyond mobility due to unavoidable thermal damage to the adjacent nerves during surgery. The question of whether benign neurogenic tumors can be palliatively resected to preserve neurological function requires further study. These results can help healthcare providers to identify individuals who may be susceptible to decreased HRQoL and develop tailored interventions to enhance their outcomes. Such interventions may include pain management strategies, psychological support, and postoperative rehabilitation programmes. Overall, our findings emphasize the need to address both tumor size and neurogenic origin when considering the long-term HRQoL of patients undergoing RATS for benign CTJ tumors, thereby providing valuable insights for healthcare professionals aiming to improve patient outcomes.

Our study had some limitations: (i) as a retrospective study, it was susceptible to selection bias. (ii) Due to the rarity of CTJ tumors, the sample size was small, which may affect the generalizability of the results. (iii) Because patients with benign tumors tend to undergo minimally invasive surgery, open surgery group’ sample size was small. (iv) The preoperative EQ-5D-5L index was not collected because this was a retrospective study.

Our study demonstrates that RATS is a safe and feasible approach for treating benign tumors of the CTJ. The choice of surgical approach should be based on the tumor location, with additional incisions used for complex cases. Clinicians should be aware of the impact of tumor size and neurogenic nature on postoperative HRQoL and develop interventions to improve outcomes for high-risk patients. In patients with tumors larger than 5 cm, careful attention should be given to the extent of wound extension during tumor removal. Preoperative evaluation and postoperative assessment of neurologic function are of utmost importance in patients with neurogenic tumors. These findings have important implications for improving the quality of care for patients with benign CTJ tumors.

### Supplementary Information

Supplementary Information.

Supplementary Video 1.

## Data Availability

The data underlying this article cannot be shared publicly due to [for the privacy of individuals that participated in the study]. The data will be shared on reasonable request to the corresponding author.

## Abbreviations

RATS: Robot-assisted thoracic surgery
CTJ: The cervicothoracic junction
HRQoL: Health-related quality of life
PBM: Preference-based measures
EQ-5D-5L: European Quality of life Group’s five-dimensional five-level questionnaire
PS: Propensity score
MO: Morbility
SC: Self-care
UA: Usual activity
PD: Pain/discomfort
AD: Anxiety/depression
