# Supplementary material for: Robot-assisted thoracic surgery for benign tumors at the cervicothoracic junction: a propensity-matched study
Source: Sci Rep. 2024 Feb 21;14:4254. doi: 10.1038/s41598-024-54653-1 (PMC10881989; doi:10.1038/s41598-024-54653-1)
Supplement: Supplementary file 1 — Supplementary Information. [file 41598_2024_54653_MOESM1_ESM.pdf]

Article Title： Robot-Assisted Thoracic Surgery for benign tumors at the cervicothoracic junction：A Propensity-Matched study

Authors： Maierhaba Maitiyasen1†, Hao Peng1†,Yvxuan Liu1, Jingfeng Li1, Chuan Gao1, Jing Chen2, Jun Yi1*

† These authors contributed equally to this work.

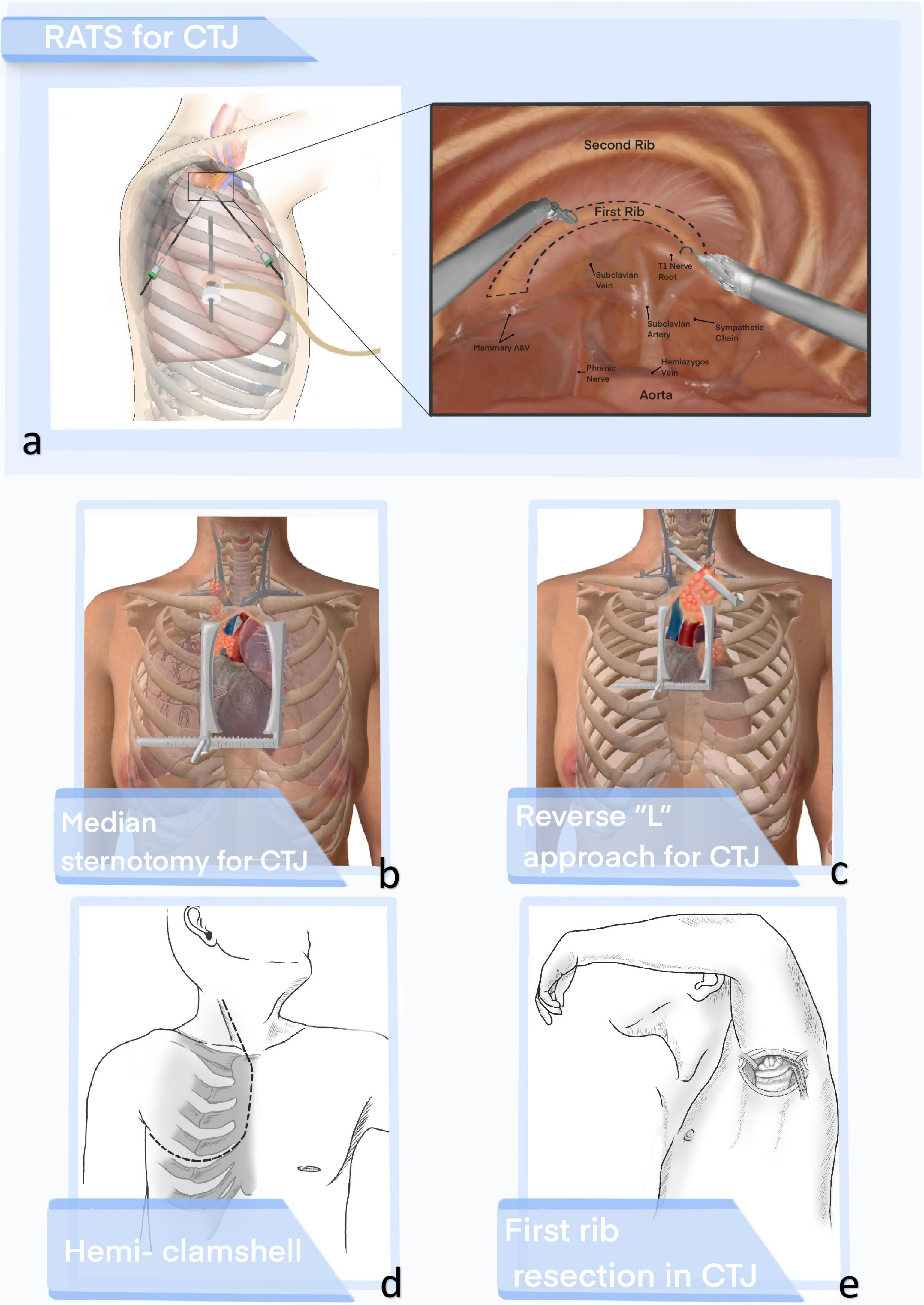
RATS for CTJ
Second Rib
First Rib
Subclavian Vein
T1 Nerve Root
Subclavian Artery
Sympathetic Chain
Mammary A&V
Phrenic Nerve
Hemiazygos Vein
Aorta
a
Median sternotomy for CTJ
b
Reverse "L" approach for CTJ
c
Hemi- clamshell
d
First rib resection in CTJ
e

Supplementary figure 1: The surgical angles provided by RATS, open sternotomy, and other commonly used surgical approaches. a: Robot-Assisted Thoracic Surgery(RATS) for Cervicothoracic Junction(CTJ) and the surgical perspectives provided by this approach. b and c: Two different open sternotomy, with the "c" incision being able to address lesions located higher up.These two open surgical approaches are now commonly used to treat tumors involving major blood vessels at the CTJ. d: The hemi-clamshell incision provides a broader field of view compared to an open sternotomy and is suitable for invasive tumors near the more distal CTJ, but it results in greater surgical trauma. e: Commonly used to treat thoracic outlet syndrome, but now the technique of using RATS to treat the first rib is also very mature.

Supplementary Table1: Clinical outcomes of benign neurogenic tumors at the CTJ : RATS versus open surgery

	RATS(N=18)	Open surgery(N=4)	P-vale
Operative Time, mean [SD] (min)	135(72.8)	219.5(76.3)	0.04*
Intraoperative Blood Loss, mean [SD] (ml)	191.1(227)	450(182.6)	0.02*
Postoperative hospital stays, mean [SD] (days)	2.72(1.01)	7.75(4.27)	0.000*
Postoperative Complication Rate (%)	22	50	0.218
Tumor size (%)			
<5cm	11(61.1)	1(25)	0.190
⩾5cm	7(38.9)	3(75)	
EQ-5D-5L index, mean [SD]	0.53(0.28)	0.70(0.08)	0.031*

*Coefficients with the statistical significance ($p < 0.05$)
